# Supplementary figures and images for: Differentially expressed genes related to plant height and yield in two alfalfa cultivars based on RNA-seq
Source: PeerJ. 2022 Oct 10;10:e14096. doi: 10.7717/peerj.14096 (PMC9558622; doi:10.7717/peerj.14096)

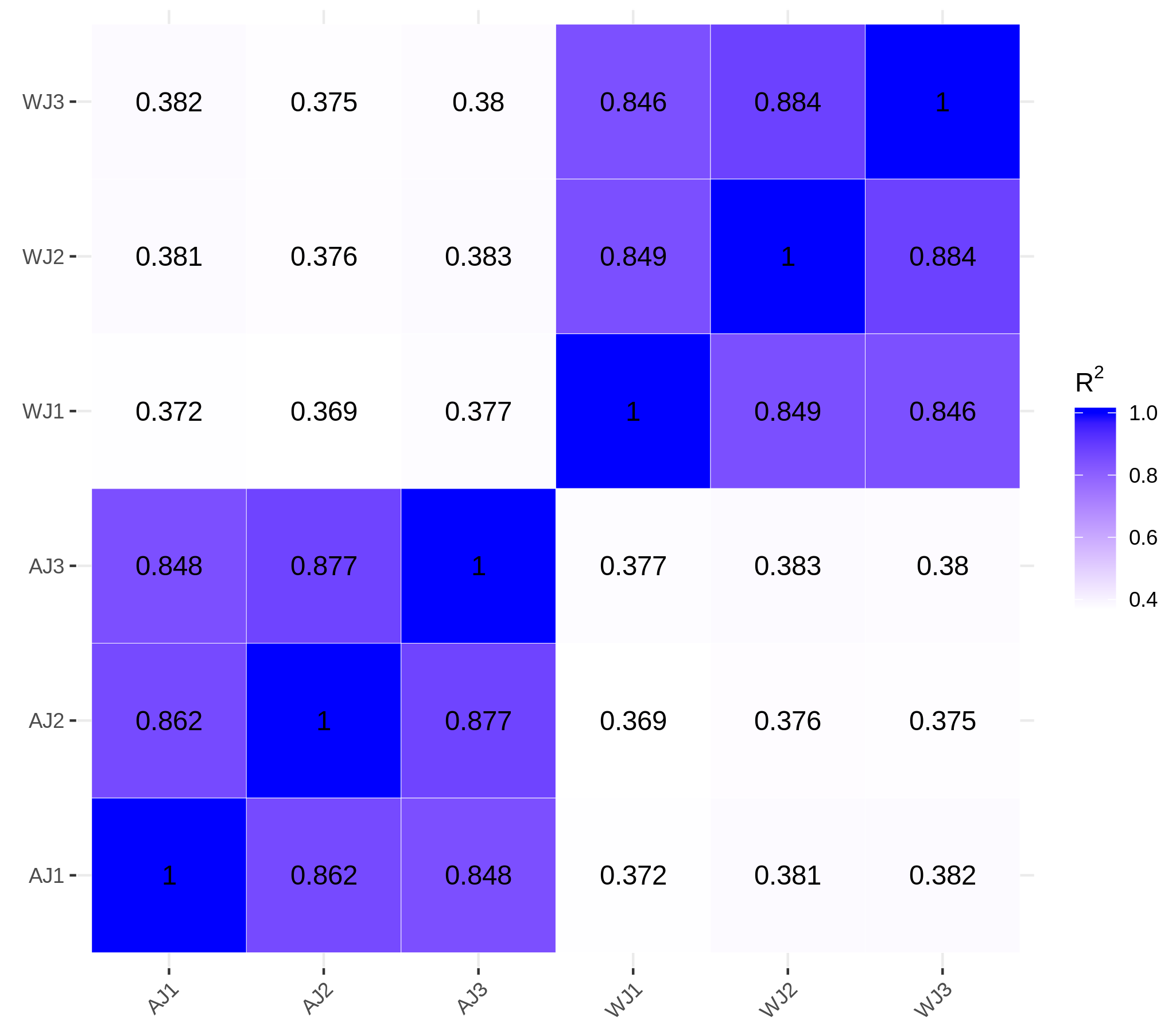

Supplement: Supplemental Information 1 [file peerj-10-14096-s001.png]

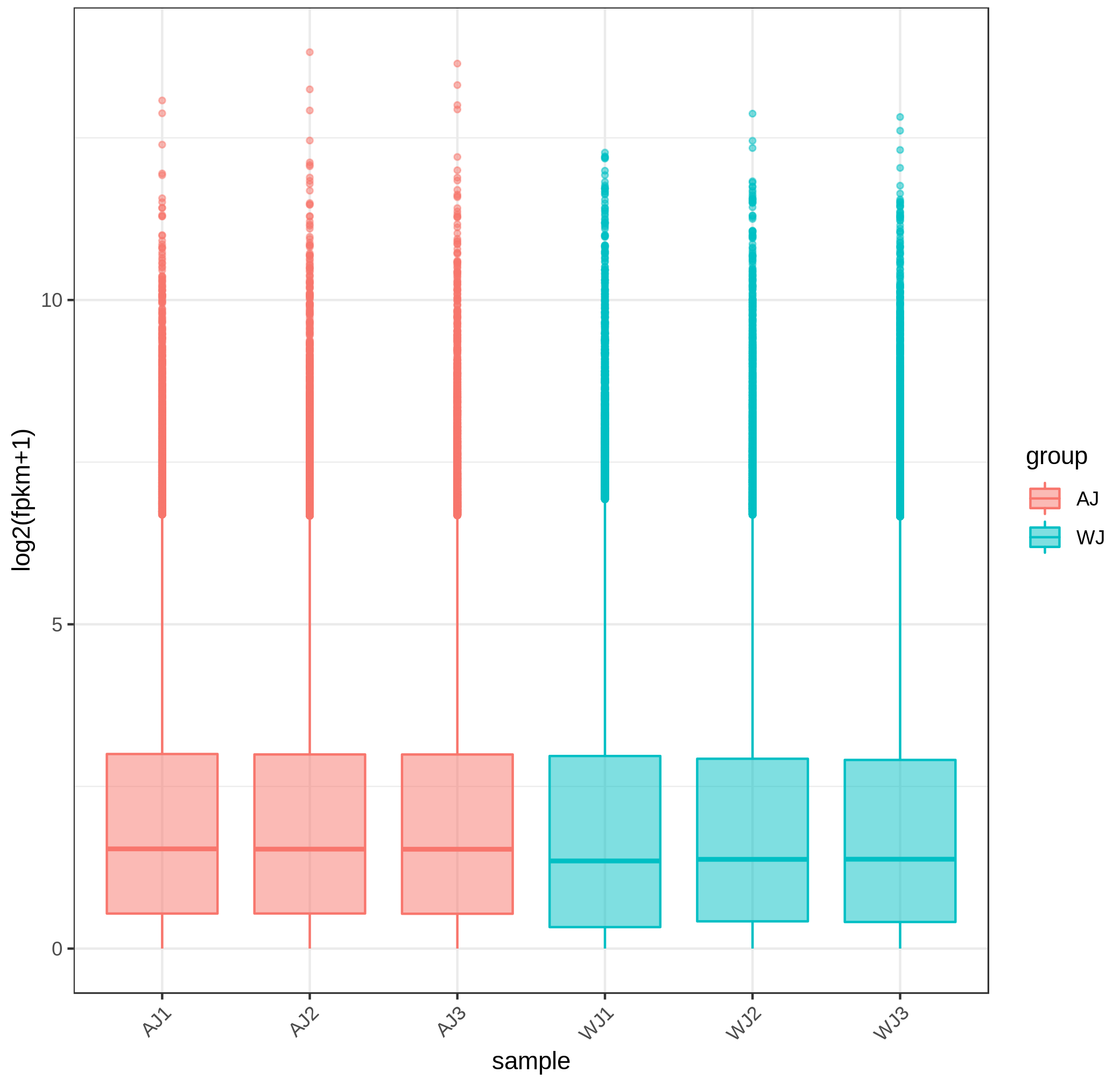

Supplement: Supplemental Information 2 [file peerj-10-14096-s002.png]
