# Supplementary material for: Differentially expressed genes related to plant height and yield in two alfalfa cultivars based on RNA-seq
Source: PeerJ. 2022 Oct 10;10:e14096. doi: 10.7717/peerj.14096 (PMC9558622; doi:10.7717/peerj.14096)
Supplement: Supplemental Information 3 [file peerj-10-14096-s003.docx]

**Table S1a Basic information of alfalfa cultivars**

| **Code** | **Varieries name** | **Fall-Dormancy** | **Source** |
| --- | --- | --- | --- |
| **1** | **WL 712** | **10.0** | **America** |
| **2** | **WL 525HQ** | **8.0** | **America** |
| **3** | **Victoria** | **6.0** | **America** |
| **4** | **Knight 2** | **2.0** | **America** |
| **5** | **Aohan** | **1.0** | **China** |

**Table S1b Physical and chemical indexes of soil before planting**

| **Index** |  |
| --- | --- |
| **available nitrogen (mg/Kg)** | 92.6 ± 0.3503 |
| **available phosphorus (mg/Kg)** | 33.2 ± 0.5300 |
| **available potassium (mg/Kg)** | 168.5 ± 0.7300 |
| **organic matteret (g/Kg)** | 12.4 ± 0.2100 |
| **pH** | 7.26 |

Different letters indicate significant difference at *P <* 0.05 among the two hormones as determined by Student’s test.

**Table S1c RNA sequenccing data statistics**

| **Summary** | **AJ1** | **AJ2** | **AJ3** | **WJ1** | **WJ2** | **WJ3** |
| --- | --- | --- | --- | --- | --- | --- |
| **Raw reads** | 45483540 | 45554284 | 47419794 | 46074794 | 45343806 | 43529196 |
| **Clean reads** | 44122812 | 44025366 | 45682478 | 44786294 | 44042592 | 42371948 |
| **Clean based** | 6.62G | 6.6G | 6.85G | 6.72G | 6.61G | 6.36G |
| **Q20 (%)** | 97.42 | 97.21 | 97.41 | 98.77 | 97.46 | 97.54 |
| **Q30 (%)** | 93.07 | 92.62 | 93.07 | 95.86 | 93.14 | 93.32 |
| **GC (%)** | 41.59 | 41.42 | 41.53 | 41.65 | 41.80 | 41.91 |
| **Error rate (%)** | 0.03 | 0.03 | 0.03 | 0.02 | 0.03 | 0.03 |
| **Mapped reads** | 40197808 | 39948756 | 41538757 | 41970046 | 40723472 | 39210630 |
| **Clean/ Raw reads (%)** | 97.01 | 96.64 | 96.34 | 97.20 | 97.13 | 97.34 |
| **Mapped/ Clean reads(%)** | 91.10 | 90.74 | 90.93 | 93.71 | 92.46 | 92.54 |
