# Supplementary material for: Differentially expressed genes related to plant height and yield in two alfalfa cultivars based on RNA-seq
Source: PeerJ. 2022 Oct 10;10:e14096. doi: 10.7717/peerj.14096 (PMC9558622; doi:10.7717/peerj.14096)
Supplement: Supplemental Information 4 [file peerj-10-14096-s004.docx]

**Table S2 Primers used for qRT-PCR**

| **Gene ID** | **Primer ID** | **Primer Seqeunce (5'to3')** | **Product length** |
| --- | --- | --- | --- |
| **MS.gene 043462** | **NAC 073 - F** | GGAGAGAATGGAATCTGCTGT | **203 bp** |
|  | **NAC 073 - R** | TTGCCACTCACGAAAACTG |  |
| **MS.gene 26389** | **NAC 010 - F** | GTTTCAAAGGTGTTTTACCAGAC | **147 bp** |
|  | **NAC 010 - R** | GTAATCCATAGGAGGAGCTTCA |  |
| **MS.gene 49710** | **NAC 081 - F** | CCGACTCAAGCGGTTCTG | **150 bp** |
|  | **NAC 081 - R** | AAGGGTCATCTTCACCATCA |  |
| **MS.gene 27204** | **WRKY 6 - F** | GTGATGGATGCCAATGGAGGA | **105 bp** |
|  | **WRKY 6 - R** | TGCGAACTGGACAACCGACT |  |
| **MS.gene 98149** | **MPK 4 - F** | GCCGCAAAGATCATCTAACTC | **249 bp** |
|  | **MPK 4 - R** | TCAAAGCATAAGCACGACC |  |
| **MS.gene 033130** | **NRP 2 - F** | ATTTCAATCCCAATCCCTAT | **158 bp** |
|  | **NRP 2 - R** | CCTTTTGTTCCCTTTCTTCTC |  |
| **MS.gene 071870** | **At2g14610 - F** | AACGGCAAACAAAGTCTCAA | **242 bp** |
|  | **At2g14610 - R** | CCTGCTGACAGAGTTCCTAATC |  |
| **MS.gene 070268** | **PER 16 - F** | TGGACCCTGTCTCACCTCAA | **205 bp** |
|  | **PER 16 - R** | CCAGTTTTAACACCAACCCTTC |  |
| **MS.gene 31033** | **WRKY 51 - F** | AGGGTGGAAAGGGATAGGGA | **141bp** |
|  | **WRKY 51 - R** | AGAAGGGTGCTGCTGTGGC |  |
| **MS.gene 035614** | **CESA 7 - F** | GGTGCTTCGTTGGGCTCT | **111 bp** |
|  | **CESA 7 - R** | GCATAGGCAAATCGCTCC |  |
| **MS.gene 007455** | **IRX 9- F** | CAAGTCATTGGATGGCATTT | **233 bp** |
|  | **IRX 9 - R** | CATAGCAAGATTTTGGAGCAGT |  |
| **MS.gene 57204** | **PER 51 - F** | CAACGCCACATAATAGAGCA | **213 bp** |
|  | **PER 51 - R** | CAAATCTTCCCAATTCTACCTC |  |
| **MS.gene 002608** | **CESA 8 - F** | CCATCGTTTACCCTTTCACAT | **180 bp** |
|  | **CESA 8 - R** | TAACACCGCTCCACCTCA |  |
|  | **GRF 6 - F** | ACCACCTGAGCGGTTATG |  |
| **MS.gene 004143** | **MPK 3 - F** | AATACGGAGACGAATGAGTTG | **155 bp** |
|  | **MPK 3 - R** | AAGGGTGGAGGAATAACATCT |  |
| **MS.gene 003286** | **POD 15 - F** | ATGAGCCAGAACAGAGGAAA | **163 bp** |
|  | **POD 15 - R** | CGCCGATGATGTTGGTGAGTT |  |
| **MS.gene 71681** | **CAD 2 - F** | GAAAGTAGCAAAGGCATTAGG | **181 bp** |
|  | **CAD 2 - F** | AGGGTGACCAACAGGAACAG |  |
| **novel. 6887** | **WRKY 22 - F** | ACAAGGCAGAAGCCATTA | **271 bp** |
|  | **WRKY 22 - R** | CAGCACTATTAGCAACCC |  |
|  | **SN 1 - R** | TGTTCCCATAAGTTCCTG |  |
| **Novel. 7725** | **TGA 1 - F** | AATGGGAGTATGTGTTTGG | **264 bp** |
|  | **TGA 1 - R** | GAGCGAGGCGTCTAAGTA |  |
|  | **GA2OX8 - R** | TTATCACCACCTTCCTTA |  |
| **MS.gene 019150** | **PIF 4 - F** | TCATCGCAAAGAAACAACG | **196 bp** |
|  | **PIF 4 - R** | TGCTTCCGCAATAACTCG |  |
|  | **β- Actin 2- F** | GATGCTGAGGATATTCAACCCC | **133 bp** |
|  | **β- Actin 2- R** | CCATGACACCAGTATGACGAGG |  |
