# Supplementary material for: Differentially expressed genes related to plant height and yield in two alfalfa cultivars based on RNA-seq
Source: PeerJ. 2022 Oct 10;10:e14096. doi: 10.7717/peerj.14096 (PMC9558622; doi:10.7717/peerj.14096)
Supplement: Supplemental Information 5 [file peerj-10-14096-s005.docx]

|  |  | **WL 712** | **WL525HQ** | **Victoria** | **Knight 2** | **Aohan** |
| --- | --- | --- | --- | --- | --- | --- |
| **Plant Height (cm)** | **August, 2019** | 72.82 ± 1.3398 ^a^ | 69.22 ± 1.3355 ^b^ | 63.50 ± 0.8968 ^c^ | 41.89 ± 0.3958 ^d^ | 41.02 ± 0.4007 ^d^ |
|  | **Mid-October, 2019** | 50.17 ± 0.3098 ^a^ | 49.65 ± 0.3060 ^ab^ | 49.20 ± 1.0536 ^b^ | 28.17 ± 0.5816 ^c^ | 28.23 ± 0.1737 ^c^ |
|  | **June, 2020** | 81.28 ± 0.9491 ^a^ | 78.21 ± 1.3904 ^b^ | 69.37 ± 0.8403 ^c^ | 43.21 ± 0.4532 ^d^ | 42.09 ± 0.4693 ^d^ |
|  | **August, 2020** | 79.41 ± 0.2840 ^a^ | 75.64 ± 1.1545 ^b^ | 68.28 ± 1.1491 ^c^ | 42.30 ± 0.4440 ^d^ | 41.60 ±0.5888 ^d^ |
| **Plant Weight (g)** | **August, 2019** | 262.6 ± 4.3935 ^a^ | 258.7 ± 5.0771 ^a^ | 258.8 ± 2.7770 ^a^ | 152.6 ± 1.8714 ^b^ | 149.2 ± 1.3198 ^b^ |
|  | **Mid-October, 2019** | 235.6 ± 1.2247 ^a^ | 232.4 ± 2.5677 ^b^ | 232.1 ± 2.1859 ^b^ | 128.5 ± 0.7085 ^c^ | 127.4 ± 0.8106 ^c^ |
|  | **June, 2020** | 329.0 ± 1.4832 ^a^ | 325.4 ± 3.6718 ^a^ | 331.2 ± 2.9215 ^b^ | 212.1 ± 0.9203 ^c^ | 211.8 ± 2.1970 ^c^ |
|  | **August, 2020** | 280.4 ± 1.4832 ^a^ | 266.6 ± 3.3126 ^b^ | 264.1 ± 1.6649 ^b^ | 152.5 ± 2.9609 ^c^ | 152.1 ± 2.1279 ^c^ |
| **Lenght of Internodde (cm)** | **August, 2019** | 7.49 ± 0.0192 ^a^ | 7.48 ± 0.0114 ^a^ | 7.50 ± 0.0158 ^a^ | 4.41 ± 0.0417 ^b^ | 4.32 ± 0.0422 ^c^ |
|  | **Mid-October, 2019** | 5.43 ± 0.0156 ^a^ | 5.27 ± 0.0167 ^b^ | 5.24 ± 0.0224 ^b^ | 2.97 ± 0.0612 ^c^ | 2.97 ± 0.0183 ^c^ |
|  | **June, 2020** | 8.13 ± 0.0559 ^a^ | 8.05 **±** 0.0705 ^a^ | 7.31 ± 0.0884 ^b^ | 4.50 ± 0.0472 ^c^ | 4.53 ± 0.0505 ^c^ |
|  | **August, 2020** | 7.87 ± 0.0678 ^a^ | 7.59 ± 0.3489 ^b^ | 7.18 ± 0.1126 ^c^ | 4.65 ± 0.0476 ^d^ | 4.60 ± 0.1641 ^d^ |
| **Stem Diameter (mm)** | **August, 2019** | 3.62 ± 0.2451 ^a^ | 3.60 ± 0.2223 ^a^ | 3.47 ± 0.0680 ^a^ | 2.05 ± 0.0277 ^b^ | 1.90 ± 0.0691 ^b^ |
|  | **Mid-October, 2019** | 2.63 ± 0.0339 ^a^ | 2.62 ± 0.0192 ^a^ | 2.60 ± 0.0228 ^a^ | 1.25 ± 0.0464 ^b^ | 1.21 ± 0.0597 ^b^ |
|  | **June, 2020** | 3.85 ± 0.0259 ^a^ | 3.62 ± 0.0167 ^b^ | 3.62 ± 0.0803 ^b^ | 2.28 ± 0.0230 ^c^ | 2.27 ± 0.0277 ^c^ |
|  | **August, 2020** | 3.83 ± 0.0295 ^a^ | 3.60 ± 0.0619 ^b^ | 3.53 ± 0.2356 ^b^ | 2.16 ± 0.0737 ^c^ | 2.00 ± 0.0432 ^c^ |

**Table S3 Growth index of different cutting times**

Different letters indicate significant difference at *P <* 0.05 among the two hormones as determined by Student’s test.
