# Supplementary material for: Differentially expressed genes related to plant height and yield in two alfalfa cultivars based on RNA-seq
Source: PeerJ. 2022 Oct 10;10:e14096. doi: 10.7717/peerj.14096 (PMC9558622; doi:10.7717/peerj.14096)
Supplement: Supplemental Information 6 [file peerj-10-14096-s006.docx]

**Table S4** **Sample comparison area statistics**

| **Sample** | **Exon** | **Intron** | **Intergenic** |
| --- | --- | --- | --- |
| **AJ1** | 4408627830 (73.4212%) | 212337727 (3.5363%) | 1383607803 (23.0426%) |
| **AJ2** | 4293006090 (71.9507%) | 220248889 (3.6914%) | 1453341991 (24.358%) |
| **AJ3** | 4516464999 (72.7953%) | 223636658 (3.6045%) | 1464235688 (23.6002%) |
| **WJ1** | 4591216083 (73.2063%) | 190342351 (3.035%) | 1490053342 (23.7587%) |
| **WJ2** | 4523812914 (74.3751%) | 181164916 (2.9785%) | 1377455188 (22.6465%) |
| **WJ3** | 4382479551 (74.8283%) | 167619577 (2.862%) | 1306613306 (22.3097%) |
